# Bioaccessibility and Biological Activities of Phytochemicals from Wild Plant Infusions and Decoctions Before and After Simulated In Vitro Digestion

**DOI:** 10.1007/s11130-025-01327-6

**Published:** 2025-02-25

**Authors:** Stefania Monari, Maura Ferri, Alessandro Zappi, Rita Escórcio, Vanessa G. Correia, André Cairrão, Artur Bento, Cristina Silva Pereira, Annalisa Tassoni

**Affiliations:** 1 Department of Biological, Geological and Environmental Sciences, University of Bologna, Bologna, Italy; 2 Department of Chemistry “Giacomo Ciamician”, University of Bologna, Bologna, Italy; 3 Instituto de Tecnologia Química e Biológica António Xavier, Universidade Nova de Lisboa, Oeiras, Portugal; 4 Interdepartmental Centre of Agri-Food Industrial Research, University of Bologna, Bologna, Italy

**Keywords:** *Borago officinalis*, *Hypericum perforatum*, Antioxidant activity, Antimicrobial activity

## Abstract

The wild plants *Borago officinalis* L. (Boraginaceae) and *Hypericum perforatum* L. (Hypericaceae) are known to have both food and medicinal uses. The aim of the work was to evaluate the bioaccessibility of potentially bioactive phytochemicals in organ-specific infusions and decoctions pre- and post- simulated in vitro oro-gastrointestinal human digestion aiming at both oral intake and topical applications. (Poly)phenols, reducing sugars, and proteins were analysed by spectroscopic and spectrometry techniques (NMR and HPLC-DAD) as well as their antioxidant and antimicrobial activities pre- and post-digestion. As expected, in vitro digestion altered the composition of the extracts. Specifically, the total amount of (poly)phenols as well as the largely correlated antioxidant activity decreased post-digestion, while reducing sugars and proteins increased. Catechins and rutin were detected in both plants. Rosmarinic acid was the most abundant compound in *B. officinalis* (on average 90% in infusions), while rutin and hyperoside in *H. perforatum* (up to 100% in stem digested extracts). *Hypericum* extracts exhibited similar activity against both Gram-positive and Gram-negative bacteria, while the *Borago* extracts showed higher activity against Gram-negative bacteria. Overall, the minimal inhibitory concentrations ranged from 0.47 to 0.94 mg/ml. In conclusion, the studied herbal preparations may provide significant amounts of bioactive compounds both pre- and post-digestion able to potentially mitigate oxidative stress-related disorders after ingestion and/or to treat skin infection diseases when applied topically.

**Supplementary Information:**

The online version contains supplementary material available at 10.1007/s11130-025-01327-6.

## Introduction

Since ancient times, humans have relied on plants for both food and medicine, passing this knowledge down through generations. A recent review on Italian ethnobotanical studies published in the last 40 years highlighted the presence of several wild and/or cultivated plants widespread all over the Italian peninsula and commonly used as foods and for medicinal purposes [1]. These included *Borago officinalis* L. (Boraginaceae) and *Hypericum perforatum* L. (Hypericaceae). The first was traditionally used in salads, soups, pancakes and pies, as well as for medicinal purposes, reportedly acting as a diuretic, laxative, and analgesic, and treating inflammation, respiratory, and skin diseases through ingestion or topical use [1, 2]. On the other hand, *Hypericum perforatum* was mainly used as a food supplement, in herbal teas and for liqueur production. Its flowers and leaves had various therapeutic applications, including treatment for acute and chronic pain, toothache, burns and rheumatisms [1, 2]. Among the reported plant preparations for both food and therapeutic purposes, water extracts like infusion and decoction were the most common both for oral intake or for topical application (for skin and infectious diseases such as burns, wounds, acne, and as cicatrizing and antimicrobial therapies) [1, 3]. The bioaccessibility of metabolites (i.e., quantity available for human absorption [4]), in raw food is altered after oral intake due to pH variations and the activity of digestive enzymes in the gastrointestinal track. Not all phytochemicals present in the digestate will become bioavailable to be absorbed by the intestinal cells and metabolized [5]. Absorption depends on their chemical characteristics, such as solubility, hydrophobicity and molecular weight [5]. Understanding how the digestion affects their availability is crucial for evaluating the properties of plant water extracts. To this extent, in vitro static oro-gastrointestinal digestion assays simulating the main digestion phases, are among the most common approaches providing insights into the bioaccessibility of food components [6, 7].Numerous phytochemicals have been isolated from traditional wild plants, including (poly)phenols, polysaccharides, and proteins [1]. (Poly)phenols have been associated with anti-inflammatory, antioxidant, antidiabetic, and anticancer effects [8]. These compounds are water-soluble, hence, their bioaccessibility depends on the efficiency of their release from plants by digestive enzymes, their degree of complexation, and the potential co-precipitation with proteins or minerals during the digestion [9, 10]. Polysaccharides include insoluble fibers that remain structurally unchanged during digestion, and others, like starch and soluble fibers, that aredepolymerised by digestive enzymes into mono-, di-, and oligo-saccharides [11], which may exhibit moderate antioxidant activity due to reducing power and radical scavenging capacity [12]. Plant proteins are also hydrolysed by intestinal into amino acids and bioavailable small molecular weight peptides, some with biological activities [13].

The study analyses the phytochemical composition and biological activity of water extracts from *B. officinalis* and *H. perforatum*. Selected plant tissues of were used to prepare infusions and decoctions, which underwent static in vitro simulated oro-gastrointestinal digestion. The research examines the bioaccessibility of major phytochemical classes [(poly)phenols, polysaccharides and proteins] as well as the antioxidant and antimicrobial activities of these extracts, both pre- and post-digestion. The findings are discussed in the context of their oral ingestion (pre- and post-digestion) or topical application (pre-digestion only), aiming at establishing a framework linking medicinal plant extracts and their phytochemical composition to specific therapeutic applications.

## Materials and Methods

This section is presented in the Supplementary Materials.

## Results and Discussion

*Borago officinalis* L. (BO) and *Hypericum perforatum* L. (HP) plants were gathered from Apulia region in Italy [2] and three different plant organs (flowers, stems and leaves) were separately collected according to previous ethnobotanical evidences [1]. Infusions and decoctions were then prepared and subjected to in vitro simulated oro-gastrointestinal digestion [14].

### Chemical Characterisation by NMR

**Fig. 1:**
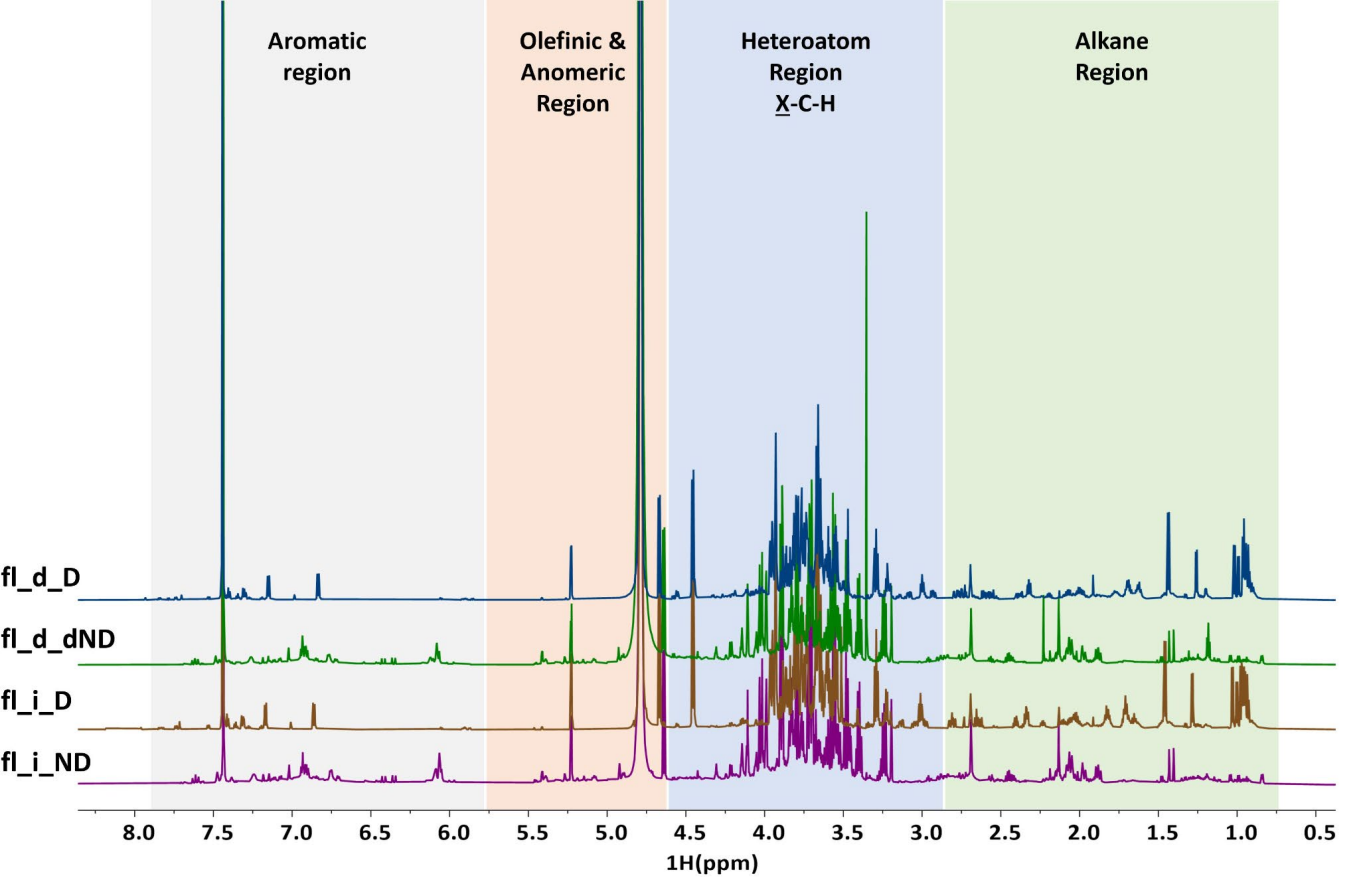
^1^H NMR spectra obtained from *Hypericum perforatum* flowers. i: infusion; d: decoction; ND: not digested; D: digested; fl.: flowers

The chemical footprint of all the extracts from either species pre- and post-digestion were analysed by NMR (Fig. 1, S1). This method allows obtaining a snapshot of the major compositional functional groups present in each extract, facilitating the identification of the samples’ similarity level. Figure 1 shows examples of ^1^H NMR spectra of HP flower infusions and decoctions before and after digestion. The spectra were divided into aromatic, olefinic and anomeric, heteroatom, and alkane regions to identify the presence of targeted classes of phytochemicals (i.e., (poly)phenols, polysaccharides and proteins). The signals detected in each region can be attributed to distinct classes of phytochemicals: aromatic region shows H associated to aromatic rings, as in many (poly)phenolics; olefinic and anomeric region is related to polysaccharides and compounds having H close to C double/triple bonds; heteroatom region (so called because H are close to other atoms besides C, like O and N) indicates esters and peptides; alkane region (aliphatic chains) identifies lipids. *Hypericum* species were reported to contain hypericin, hyperoside, hyperforin, phenolic acids and flavonoids, fatty acids, terpenes and terpenoids [15, 16]; whereas *Borago* species contain (poly)phenolic and flavonoid compounds, fatty acids, and terpenes [17, 18].

**Fig. 2:**
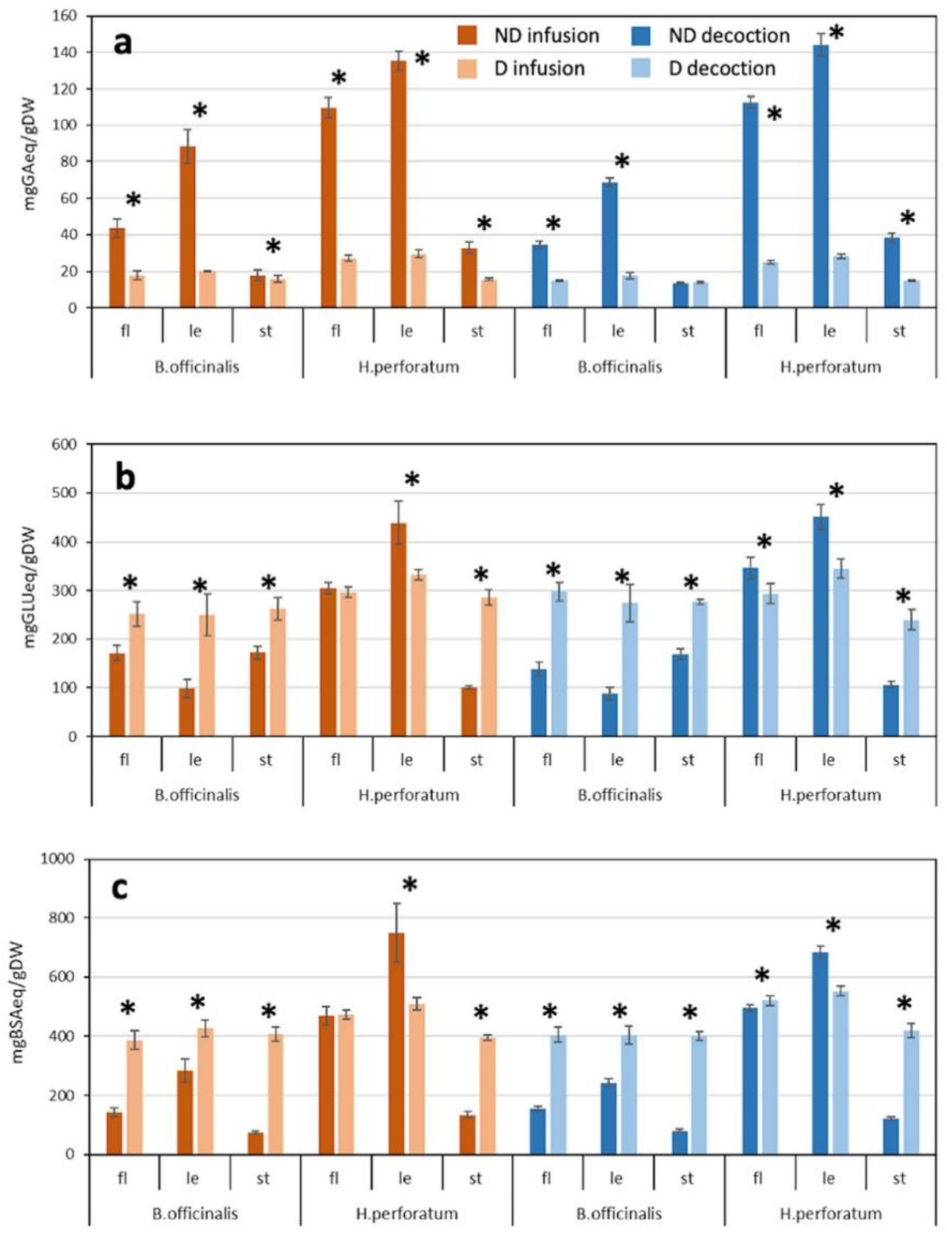
Total amounts of (**a**) (poly)phenols, (**b**) reducing sugars, (**c**) proteins of aqueous extracts of *Borago officinalis* and *Hypericum perforatum* detected by spectrophotometric analysis. ND: not digested; D: digested; fl.: flowers; le: leaves; st: stems; GA: gallic acid, GLU: D-glucose, BSA: bovine serum albumin, eq: equivalent, DW: dry weight. Star symbol (*) indicates statistically significant differences (*p* < 0.05, *t*-test) between not digested and digested samples. Data are the mean ± SD (*n* = 3)

**Fig. 3:**
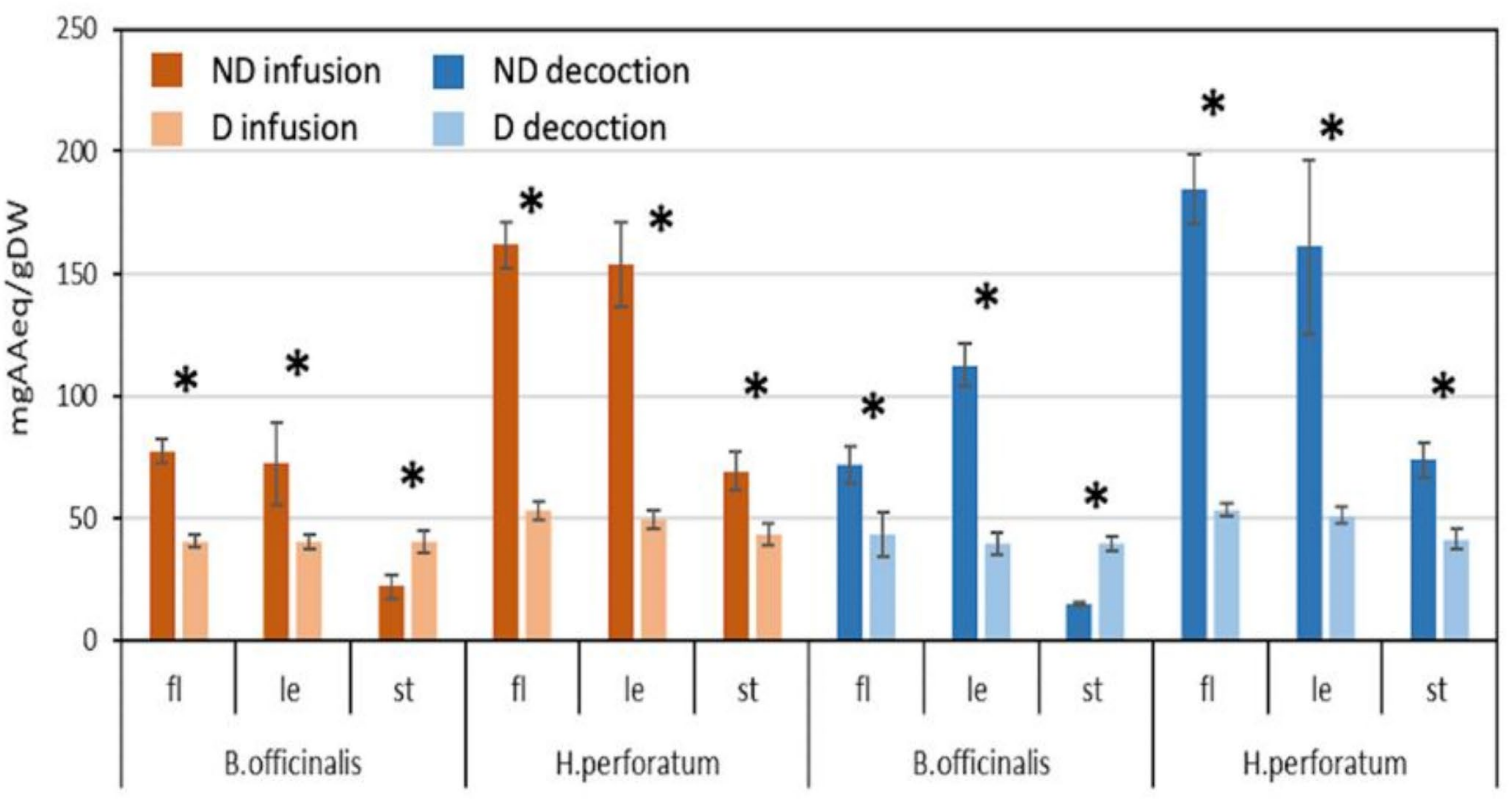
Antioxidant activity of aqueous extracts of *Borago officinalis* and *Hypericum perforatum* after spectrophotometric analysis. ND: not digested; D: digested; fl.: flowers; le: leaves; st: stems; AA: ascorbic acid, eq: equivalent, DW: dry weight. Star symbol (*) indicates statistically significant differences (*p* < 0.05, *t*-test) among not digested and digested samples. Data are mean ± SD (*n* = 3)

No specific (poly)phenols were identified in the spectra, likely due their concentration being below the NMR detection level. Given the complex footprint of the extracts, rather than putatively assigning signals to specific compounds, all spectra were compared using PCA analysis (Fig. S1). The PCA graph shows sample dispersity, with digestion clearly separating them into two clusters (PC1): pre- and post-digested extracts. The latter exhibit high similarity, confirming that digestion effectively altered the phytochemical composition in agreement with the known breakdown and modification of (poly)phenols, protein and polysaccharides [9, 10, 19] and with the following analyses (Figs. 2 and 3). The extracts post-digestion grouped (PC2) according to plant organ rather than to the species or the extraction method with no difference between infusion or decoction samples (Fig. S1). The PCA loadings analysis, based on correlation analysis, revealed significant heterogeneity among samples. No single NMR signal accounted for their dissimilarity; instead, variation was distributed across all NMR regions, indicating a complex and multifaceted pattern. Different conclusions were obtained in an NMR-based study where infusions and decoctions from whole aerial parts of 10 different herbs could be discriminated [20]. PCA analysis on pre-digestion samples revealed three clusters, primarily reflecting plant tissue rather than the extraction method (Fig. S1). One cluster includes all leaf extracts (with those from HP flowers as outliers), another contains the extracts from BO flowers and HP stems, and the third the extracts from BO stems. This suggests that the phytochemical NMR profiles of leaves from both species are similar, with the most distinct profiles coming from BO stem extracts. The undigested BO extracts are scattered in the graph, clearly separated by plant tissue: flowers, leaves and stems. These results are consistent with previous data on traditional medicinal plants, where different plant organs showed distinct spectral patterns [21, 22].

### Spectrophotometric Analysis

To characterize the collected extracts, the levels of (poly)phenols, polysaccharides, and proteins in infusions and decoctions of each plant tissue before and after digestion, were analysed spectrophotometrically (Fig. 2). Before digestion, infusion and decoction of leaves of both species showed the highest total (poly)phenol contents (135.3 and 144.1 mg GAeq/gDW in HP and 88.3 and 68.7 mg GAeq/gDW in BO, respectively) followed by flowers and stems (Fig. 2a). A significant decrease in (poly)phenols was observed post-digestion in all extracts (*p* < 0.05), except for BO stem decoctions. This reduction was, on average, -57 and − 80%, respectively in both extracts of flowers and leaves for both species. Stem samples from HP showed as well high (poly)phenol reduction after digestion (-52% and − 61% in infusion and decoction, respectively). BO stem extracts only exhibited a minor reduction (-11%) in infusion (Fig. 2a). These results are consistent with the reported reduction of (poly)phenols after simulated digestion in tea infusions [23]. Previous studies have reported that digestion alters the bioaccessibility and concentration of (poly)phenols [9], mainly due to the effects of gastrointestinal pH and/or proteolytic enzymes [10, 24].

In contrast to (poly)phenols, the levels of reducing sugars in extracts of both species generally increased after digestion, with the largest increases observed in BO leaf extracts (on average + 154.4% in infusion; +212.5% in decoction) and HP stems extracts (+ 185.8% in infusion; +125.5% in decoction) (Fig. 2b). This was attributed to the polysaccharide breakdown due to pH changes, digestive enzymes activity, and presence of salts in the simulated digestion fluids in accordance with literature [19]. Exceptions to the above-mentioned trend were the undigested extracts of HP leaves and flowers that were 1.3-fold and 1.2-fold higher, respectively, than their digested counterparts (Fig. 2b). Consistent with these findings, previous publications reported both increasing and decreasing polysaccharide amounts in different vegetable matrices subjected to simulated digestion process. For example, levels increased in digestates of *Aloe vera* (L.) Burm.f. leaves, remained unchanged in squash and carrot polysaccharides resistant to degradation, and decreased in *Opilia amentacea* Roxb. fruits [11]. Similarly to polysaccharides, the protein levels in general greatly increased after digestion with some exceptions (Fig. 2c). In fact, the highest level of proteins pre-digestion was observed in HP leaf extracts (749.1 and 683.2 mg BSAeq/gDW in infusions and decoctions, respectively), with a reduction of about 25.6% post-digestion (508.6 and 551.9 mg BSAeq/gDW in infusions and decoctions). On the other hand, the highest increase in protein content post-digestion was detected in stem extracts of both species (on average + 419.2% and + 218.5% for BO and HP, respectively). This increase reflects the action of the pancreatin enzymatic cocktail that includes trypsin, amylase, and lipase able to hydrolyse proteins, starch and fats [25]. Studies on in vitro simulated digestion of protein sources such as sorghum, black beans, and whey, have shown no intact proteins after hydrolysis [26]. In agreement with the literature, present results indicate that proteins were broken down into smaller peptide fragments, resulting in higher contents in nearly all post-digestion samples (Fig. 2c).

### (Poly)phenols Characterization by HPLC-DAD

The detailed (poly)phenolic composition of *B. officinalis* and *H. perforatum* pre- and post-digestion extracts was evaluated by HPLC-DAD characterization (Table 1). Overall, the extracts from flowers and leaves were richer in (poly)phenols compared to those from stems. In BO extracts (Table 1) two flavanols (catechin and epicatechin), one phenolic acid (gallic acid), two hydroxycinnamic acids (ferulic and rosmarinic acids) and one flavonol (rutin), were identified.

**Table 1:** HPLC-DAD (poly)phenols quantification of *Borago officinalis* L. and *Hypericum perforatum* L. not digested (ND) and digested (D) infusions and decoctions

Preparation	Organ	Samples	CAT (mg/gDW)	EC (mg/gDW)	EGC (mg/gDW)	CAFA (mg/gDW)	RUT (mg/gDW)	RUT2 (mg/gDW)	HYPS (mg/gDW)	Total (poly)phenols (mg/gDW)
***Hypericum perforatum*** ** L.**										
Infusion	Flowers	ND	-	5.0 ± 0.45	-	1.22 ± 0.32	20.99 ± 1.25	11.36 ± 1.14	12.66 ± 0.75	51.25 ± 2.60^a^
		D	0.21 ± 0.05	0.38 ± 0.21	-	0.07 ± 0.03	2.73 ± 0.32	1.45 ± 0.19	1.65 ± 0.19	6.49 ± 0.89^b^
	Leaves	ND	-	2.88 ± 0.29	-	3.01 ± 0.54	30.81 ± 4.77	12.44 ± 2.07	18.59 ± 2.88	67.73 ± 10.03^a^
		D	-	-	-	0.14 ± 0.04	3.37 ± 0.21	1.29 ± 0.06	2.03 ± 0.12	6.84 ± 0.33^b^
	Stems	ND	0.25 ± 0.02	1.54 ± 0.07	1.95 ± 0.08	0.09 ± 0.01	3.53 ± 0.09	1.57 ± 0.05	2.13 ± 0.06	11.07 ± 0.15^a^
		D	-	-	-	-	0.36 ± 0.10	0.18 ± 0.04	0.21 ± 0.06	0.75 ± 0.19^b^
Decoction	Flowers	ND	2.89 ± 0.07	6.63 ± 0.40	-	1.11 ± 0.07	23.21 ± 1.83	10.37 ± 1.57	14.00 ± 1.11	58.22 ± 3.83^a^
		D	0.17 ± 0.07	0.22 ± 0.06	-	0.05 ± 0.02	2.30 ± 0.13	1.00 ± 0.08	1.39 ± 0.08	5.12 ± 0.15^b^
	Leaves	ND	-	2.99 ± 0.34	-	2.62 ± 0.53	30.76 ± 0.83	11.66 ± 0.73	18.56 ± 0.50	66.59 ± 2.44^a^
		D	-	-	-	0.11 ± 0.03	2.98 ± 0.13	1.06 ± 0.05	1.80 ± 0.08	5.95 ± 0.17^b^
	Stems	ND	0.22 ± 0.01	1.30 ± 0.12	1.30 ± 0.07	0.06 ± 0.01	3.11 ± 0.18	1.45 ± 0.14	1.88 ± 0.11	9.31 ± 0.59^a^
		D	-	-	-	-	0.30 ± 0.06	0.15 ± 0.02	0.18 ± 0.03	0.63 ± 0.11^b^
***Borago officinalis*** ** L.**										
**Preparation**	**Organ**	**Samples**	**GALA (mg/gDW)**	**CAT (mg/gDW)**	**EC** **(mg/gDW)**	**FERA (mg/gDW)**	**RUT (mg/gDW)**	**ROSMA (mg/gDW)**		**Total** **(poly)phenols** **(mg/gDW)**
Infusion	Flowers	ND	-	0.02 ± 0.01	0.06 ± 0.01	0.09 ± 0.03	0.05 ± 0.01	2.94 ± 0.90		3.15 ± 0.95^a^
		D	0.03 ± 0.00	-	-	-	-	0.10 ± 0.06		0.13 ± 0.06^b^
	Leaves	ND	-	-	0.13 ± 0.05	0.26 ± 0.09	-	10.97 ± 2.12		11.36 ± 2.25^a^
		D	0.03 ± 0.00	-	-	-	-	0.82 ± 0.07		0.85 ± 0.07^b^
	Stems	ND	-	-	-	-	-	2.49 ± 1.28		2.49 ± 1.28^a^
		D	0.03 ± 0.00	-	-	-	-	0.11 ± 0.07		0.15 ± 0.07^b^
Decoction	Flowers	ND	-	0.02 ± 0.00	0.07 ± 0.01	0.04 ± 0.00	0.04 ± 0.01	0.99 ± 0.13		1.17 ± 0.15^a^
		D	0.03 ± 0.00	-	-	-	-	0.05 ± 0.01		0.08 ± 0.01^b^
	Leaves	ND	-	-	0.11 ± 0.04	0.20 ± 0.06	-	5.41 ± 0.85		5.72 ± 0.90^a^
		D	0.02 ± 0.01	-	-	-	-	0.30 ± 0.06		0.32 ± 0.07^b^
	Stems	ND	-	-	-	-	-	0.04 ± 0.02		0.04 ± 0.02^a^
		D	0.03 ± 0.00	-	-	-	-	-		0.03 ± 0.00^b^

Data are the mean ± SD (*n* = 3). Different letters (a, b) indicate statistically significant differences (*p* < 0.05, *t*-test) among total identified (poly)phenol levels of not digested (ND) and digested (D) samples. CAT: catechin; EC: epicatechin; EGC: epigallocatechin; CAFA: caffeic acid; RUT: rutin; RUT2: rutin isomer; HYPS: hyperoside; GALA: gallic acid; FERA: ferulic acid; ROSMA: rosmarinic acid; DW: dry weight

The presence of these compounds in BO extracts was previously demonstrated [27], with the exception of catechins, which were here identified in undigested infusions and decoctions. Rosmarinic acid showed the highest concentration, being on average 90% and 87% of the detected compounds respectively in infusions and decoctions. These results are in agreement with early studies in which rosmarinic acid was consistently present in different organs of BO [18, 27]. This compound is considered the strongest antioxidant among all hydroxycinnamic acid derivatives, and also exhibits antidiabetic, antimicrobial, anti-inflammatory, cardioprotective and hepatoprotective activities [28], thereby supporting therefore the broad traditional therapeutic use of BO [1]. In HP infusions and decoctions (Table 1), three flavanols (catechin, epicatechin, and epigallocatechin), one hydroxycinnamic acid (caffeic acid) and two flavonols (rutin and hyperoside) were identified. With the exception of epigallocatechin, all compounds were previously reported to be abundant in HP aerial tissues [29]. A second peak (RUT2) with similar spectrum and the maximum absorbance wavelength (365 nm) of rutin (RUT) but different retention time, was detected in all the extracts and was putatively identified as a rutin structural isomer (RUT2). Rutin and hyperoside (HYPS), both derived from quercetin, were the most abundant (poly)phenols in HP extracts (Table 1). Quercetin-derivatives have been associated with a wide range of biological activities, in particular neuroprotective properties for instance against cerebral ischemia, Parkinson and Alzheimer’s diseases [30]. In agreement with spectrophotometric analysis data (Fig. 2a), the concentration of (poly)phenolic compounds decreased after digestion (Table 1). In digested HP extracts, caffeic acid decreased reaching an average concentration of 0.24 mg/gDW in infusions and decoctions (Fig. 3a), while rutin (RUT + RUT2) decreased on average by 89%. In BO extracts, rosmarinic acid decreased by an average of 95% after digestion, while ferulic acid was no longer detectable in digestates. Similar results reporting the reduction of (poly)phenols after oro-gastrointestinal digestion, likely due to their degradation or transformation, were previously reported [9, 24]. On the other hand, after digestion, gallic acid levels increased in BO samples probably due its release from (poly)phenolic gallate-forms which are very abundant in plants.

### Antioxidant and Antimicrobial Activities

As expected, antioxidant activity results showed a significant reduction in antioxidant activity in most HP extracts after digestion (Fig. 3) while undigested HP flowers and leaves extracts displayed the highest levels (161.9 and 158.8 mg AAeq/gDW in infusions, 184.6 and 160.9 mg AAeq/gDW in decoctions, respectively). After digestion, the activity decreased by an average of 69%, with lower levels detected in BO samples, except for the leaf decoction (about *−* 65%). A similar decline in antioxidant activity after digestion has been observed in various plant preparations, including herbal teas [23].

The antioxidant properties of plant-based food and beverages are primarily attributed to (poly)phenolic compounds [10]. The total (poly)phenol contents and antioxidant activity of extracts of both plant species were positively correlated, both before (R^2^ = 0.91) and after digestion (R^2^ = 0.86). This suggests that (poly)phenolic compounds were likely the major contributors to the observed antioxidant activity as demonstrated previously for HP and BO extracts [16, 27, 31]. Nonetheless, the potential contribution of reducing sugars [12] (which increase post-digestion, Fig. 2b) and peptides (Fig. 2c) cannot be entirely disregarded.

Since the analysed extracts were traditionally used in topical applications against skin infections and wounds [1, 3], undigested extracts were also tested for their broad antimicrobial activity. Initially, the inhibitory effect of each undigested extract (from 15 to 0.03 mg/ml) was tested against two model bacteria: *Staphylococcus aureus* (Gram-positive) and *Escherichia coli* (Gram-negative). *S. aureus* is a major cause of both mild skin infections (e.g., furuncles, abscesses and of wounds) and invasive infections affecting respiratory and cardiovascular systems [32]. *E. coli* is generally a harmless inhabitant of the gastrointestinal tract, but can cause infections of urinary tract, skin and bloodstream, and meningitis [33]. The minimum inhibitory concentration (MIC) (i.e., the lowest sample concentration inhibiting bacterial the growth), was determined for each not digested extract (Table S1). HP samples exhibited similar activity against both Gram-positive and Gram-negative bacteria with the exception of leaves decoction, whereas BO samples were consistently more active against the Gram-negative *E. coli*. The lowest MIC values against *E. coli* were observed for the flower decoctions of both species (HP 0.94 mg/ml; BO 0.47 mg/ml) and for infusions of BO leaves (0.94 mg/ml). Against *S. aureus*, the most effective extracts were HP leaf infusion/decoction and HP flower decoction (all at 0.94 mg/ml) (Table S1). Infusions and decoctions of different plant tissues often exhibited varying MICs against the same bacterium. This aligns with the extracts’ complex, multicomponent nature, as shown by the characterization analyses (Figs. 1 and 2; Table 1). Additionally, after 24 h of exposure to infusions or decoctions, bacterial metabolic activity rate (high in living/dividing cells or low in stressed/dead cells) was measured by the MTT assay (Table S2) [34]. The MTT data generally were in consistent with the measured cell optical density (OD at Abs_600nm_) (Table S2). To confirm these findings. bacteria cultures exposed to the lowest MIC concentrations (Table S1) were plated to determine the colony-forming units (CFUs) (Fig. S2). As expected, the growth inhibition (GI) reached nearly 100% for all extracts (Fig. S2), except for the HP flower decoctions and leaf infusions against *E. coli* that reached about 80% GI (Fig. S2a) indicating bacteriostatic rather than bactericidal activity. In agreement with present data, previous studies reported HP extracts to inhibit the growth of both bacteria [35] or, alternatively, only of *S. aureus* [31]. The detected antimicrobial activity of HP extracts could be attributed to the presence of (poly)phenolic compounds, such as flavonoids (Figs. 1 and 2; Table 1). Consistent with previous reports [18], BO water extracts showed higher antimicrobial activity against Gram-negative bacteria compared to Gram-positive (Table S1), which may be related to the presence of flavonoids and other (poly)phenols (Figs. 1 and 2; Table 1). The antioxidant and antimicrobial activities of pre- and post-digestion infusions and decoctions from different organs of HP and BO (Fig. 3 and S2, Tables S1-S2) are linked to their traditional uses as food and medicine [1]. The present results confirmed that, after water extraction and in vitro digestion (simulating oral intake), both species retained, or even enhanced, significant antioxidant properties, which may contribute to the traditionally observed health benefits. Additionally, the observed antimicrobial activities could support their traditional use against infections, both internally or on the skin. To better understand these health benefits for humans, further investigation is needed on phytochemical variability (both species are wild plants), individual human responses, and the interactions with the gut microbiome.

## Conclusions

Food and medicinal plants contain numerous bioactive compounds, but their health effects depend on digestion metabolism. Analyzing raw extracts may not predict in vivo effects once ingested, as bioaccessibility of phytochemicals changes in the oro-gastrointestinal tract. This study assessed the impact of digestion on the bioaccessibility of (poly)phenols, reducing sugars, and proteins in *H. perforatum* and *B. officinalis* infusions and decoctions using a simulated in vitro digestion. Post-digestion, (poly)phenol bioaccessibility and antioxidant activity decreased, while reducing sugars and proteins increased. Undigested extracts showed significant antimicrobial activity against Gram-positive and Gram-negative bacteria, supporting traditional topical uses and suggesting new applications for skin infections. This first systematic study correlating bioactivity and phytochemical composition of these herbal preparations, highlights their therapeutic potential and lays the groundwork for further biochemical and pharmacological research.

## Electronic Supplementary Material

Below is the link to the electronic supplementary material.

Supplementary Material 1

## Data Availability

No datasets were generated or analysed during the current study.
