# Supplementary material for: Bioaccessibility and Biological Activities of Phytochemicals from Wild Plant Infusions and Decoctions Before and After Simulated In Vitro Digestion
Source: Plant Foods Hum Nutr. 2025 Feb 25;80(1):81. doi: 10.1007/s11130-025-01327-6 (PMC11861409; doi:10.1007/s11130-025-01327-6)
Supplement: Supplementary file 1 — Supplementary Material 1 [file 11130_2025_1327_MOESM1_ESM.docx]

**Supplementary Material**

**Title:** Bioaccessibility and biological activities of phytochemicals from wild plant infusions and decoctions before and after simulated *in vitro* digestion

**Journal name:** Plant Foods for Human Nutrition

**Authors:** Stefania Monari^a^, Maura Ferri^a^, Alessandro Zappi^b^, Rita Escórcio^c^, Vanessa G. Correia^c^, André Cairrão^c^, Artur Bento^c^, Cristina Silva Pereira^c^, Annalisa Tassoni^a,d*^.

^a^ Department of Biological, Geological and Environmental Science, University of Bologna, Italy

^b^ Department of Chemistry "Giacomo Ciamician", University of Bologna, Italy

^c^ Instituto de Tecnologia Química e Biológica António Xavier, Universidade Nova de Lisboa, Oeiras, Portugal

^d^ Interdepartmental Centre of Agri-Food Industrial Research, University of Bologna, Italy

*Corresponding author: [annalisa.tassoni2@unibo.it](about:blank)

**Materials and Methods**

**Plant materials and sample preparation**

*Borago officinalis* L. (BO) and *Hypericum perforatum* L. (HP) plants from Apulia region (Italy) were harvested in Gargano area (41°51'39.2″ N, 15°21'03.4″ E) [1] and their flowers, leaves and stems were separated. Voucher specimens were collected and deposited in the Herbarium of the University of Bologna with the following reference numbers: BO, BOLO602031; HP, BOLO602032.

Each tissue-type sample was frozen in liquid nitrogen, lyophilised and grinded by means of a blender. Infusions and decoctions of each plant grinded tissue were prepared adding always 20 ml of distilled water to 0.4 g of sample; for infusions boiling water was used, then kept 5 min at room temperature; for decoctions water at room temperature was used, then boiled 5 min. The water extracts were centrifuged (4500 *g*, 4°C, 10 min), the supernatants recovered and stored at -20°C until further analyses or *in vitro* digestion. Aliquots of infusions and decoctions from either species, pre- and post-digestion, were lyophilized (Heto PowerDry PL3000; condenser temperature -55°C, automatic vacuum release, no pressure regolation) for NMR characterisation.

***In vitro* oro-gastrointestinal digestion**

The simulated *in vitro* oro-gastrointestinal digestion was performed by applying the protocol of the static model system INFOGEST [2], which mimics the oral, gastric and small intestinal phases of human digestion process. Firstly, the number of activity units of each enzyme used in the protocol (*i.e.,* α-amylase, pepsin and pancreatin) was determined [2]. The simulated digestion protocol was applied to 3 ml of infusions or decoctions. The samples were diluted 1:1 (v/v) with simulated salivary fluid and α-amylase (human salivary α-amylase, Sigma-Aldrich, 75 U/ml) and incubated 2 min (oral phase). Then, the simulated gastric fluid was added to oral bolus to achieve a final ratio of 1:1 (v/v), the pH was adjusted to 3 (with HCl 1M), added with pepsin (Sigma-Aldrich, 2000 U/ml) and incubated 2 h (gastric phase). Finally, the simulated intestinal fluid was added to gastric chyme to achieve a final ratio of 1:1 (v/v), the pH was adjusted to 7.0 (with NaOH 1M), added with pancreatin (from porcine pancreas, trypsin activity, 100 U/ml) and incubated for 2 h (intestinal phase). All the incubations were performed at 37°C with shaking (130 rpm). At the end of the intestinal phase, samples were immediately stored in ice to stop enzymatic reactions, then frozen at -20°C until analysis.

**NMR baseline characterisation**

Nuclear magnetic resonance (NMR) spectroscopy was used to obtain a snapshot of the compositional functional groups of the plant extracts, with detailed structural related features such as bonds between molecules (macromolecular view). The lyophilized infusions and decoctions (5 mg of each) pre- and post-digestion, were dissolved in 500 μl of D_2_O. The NMR spectra (^1^H, ^1^H–13C HSQC, ^1^H–13C HSQC-TOCSY) were recorded using an Avance III 800 MHz CRYO (Bruker Biospin, Rheinstetten, Germany) and acquired in deuterium oxide (D_2_O) using 5 mm diameter NMR tubes, at 25 °C. MestReNova, Version 11.04-18998 software (Mestrelab Research, S.L.) was used to process the acquired raw data. The acquired ^1^H NMR spectra were processed using the statistical tool Rnmr 1D package, which categorizes them based on the degree of similarity [3]. All spectra were first aligned, baseline correction was applied and the segments [8.0;7.46]; [7.43;4.90]; [4.70;.2.71]; [2.68;0.5] ppm analysed. The segments associated to solvents were deleted.

**Spectrophotometric analyses**

Infusion and decoction samples pre- and post-digestion were analysed to assess total amounts of phenols (Folin-Ciocalteu method [4]), reducing sugars (3,5-dinitrosalicylic acid (DNS) method [5]), and proteins (Lowry et al. [6]), and the antioxidant activity, ABTS (2,20-azino-di-(3-ethylbenzthiazoline sulfonic acid)) method [4] . All methods used external standards, respectively gallic acid (GA), D-glucose (GLU), bovine serum albumin (BSA) and ascorbic acid (AA), by means of dose-response calibration curves: 0 - 15 µg for GA; 50 - 500 µg for GLU; 0 – 200 µg for BSA, and 0 - 2 µg for AA. The results were expressed as mg of equivalent standard per g of dry weight (mg eq/gDW).

**Phenols characterization by HPLC-DAD**

To recover phenols in the plant extracts pre- and post-digestion, 1 ml of infusion/decoction was loaded into a solid-phase Strata-X column (33 μm polymeric sorbent, 60 mg / 3 ml, Phenomenex, Torrence, CA, USA); phenols were eluted with 100% (v/v) methanol, dried and resuspended in 200 μl of 1:9 acetonitrile 100%/water 99.8%:0.2% acetic acid (v/v). The extracts were injected into the HPLC-DAD (column Gemini C18, 5 μm particles 250 x 4.6 mm, pre-column Security- Guard Ea, Phenomenex, Torrence CA, USA) equipped with an on-line diode array detector (MD-2010, Plus, Jasco Instruments, Großumstad, Germany) [7]. This HPLC-DAD separation procedure processes simultaneous the analysis of 25 compounds by means of a 45 min dynamic gradient [8]. The HPLC standards were purchased from Sigma-Aldrich (Milano, Italy). For each phenolic sample, five chromatograms obtained at different wavelengths were analysed to quantify single compounds, namely at 270 nm - gallic (GA), protocatechuic (PROTA), syringic (SIRA), vanillic (VANA) and trans-cinnamic (CINA) acids, epigallocatechin (EGC), catechin (CAT), epicatechin (EC), epigallocatechin-gallate (EGCG), and vanillin (VAN); at 285 nm - naringenin (NAR); at 305 nm - p-coumaric acid (COUMA); at 323 nm - chlorogenic (CHLORA), caffeic (CAFA), trans-ferulic (FERA), sinapic (SINA), rosmarinic (ROSMA) acids, apigenin (API) and piceatannol (PICEAT); rutin (RUT), myricetin (MYR), quercetin (QUERC), kaempferol (KAE), luteolin (LUT); and at 365 nm - luteolin-7-glucoside (LUT-7-glu).

**Antimicrobial activity assays**

Stock solutions of each plant dried extract were prepared (30 mg/ml in water). *S. aureus* NCTC8325 and *E. coli* TOP 10 bacterial strains were grown in Mueller-Hinton broth (MHB) (Merck KGaA, Darmstadt, Germany) overnight at 37°C. Aliquots of 100 μl of bacteria suspension were added to 96-well microplates containing 2-fold serial dilutions in water (100 μl, concentrations ranging from 15 to 0.03 mg/ml) of the stock solutions to obtain a cell density of 10^5^ cells/ml in each well. The microplates were incubated for 24 h at 37 °C, and the optical density at 600 nm (OD Abs_600nm_) was measured to evaluate bacterial growth (Infinite M nano+ Tecan microplate reader). Wells with only media (abiotic), bacteria (biotic), and extracts (negative) were utilized as controls. Minimal Inhibitory Concentration (MIC) was defined as the lowest concentration of a compound at which bacterial growth was inhibited at least of 70%. After 24h of incubation with the plant extracts, 100 μl of each well solution were transferred to a new plate and 10 μl of MTT (thiazolyl blue tetrazolium bromide) (0.025 g in 25 ml Phosphate Buffered Saline buffer (PBS)) were added and incubated at 37°C in the dark. After 30 min, 100 μl of 10% (w/v) sodium dodecyl sulphate (SDS) followed by 25 μl of 37% HCl were added to each well to solubilize formazan crystal produced in the solution, and the plate incubated 2h at room temperature in the dark. The sample absorbance was measured at 560 nm and 700 nm and the values were subtracted to obtain the final results (Abs_560_ – Abs_700_). Microbial growth inhibition (GI) percentage was calculated relatively to the biotic control. Previous sample concentrations showing the lowest MIC values and inducing at least 70% of bacteria growth inhibition, were selected to assess bacterial cells’ viability. After 24h incubation at 37°C, bacterial cells were plated on Petri dishes containing Mueller Hinton Agar (Merck KGaA, Darmstadt, Germany). After 24 h at 37°C, the number of distinct growing colony forming units (CFUs) was counted. The percentage of growth was calculated as follows respect to the biotic control:

$$\text{\% growth =}\left( \frac{\left( {CFUs}_{sample} \right)-\left( {CFUs}_{control} \right)}{{CFUs}_{control}} \right)x 100$$

In other words, the percentage of growth was quantified as the difference between the number of colonies forming units of sample and biotic control, divided by the number of control colony forming units and multiplied by 100.

**Statistical analyses**

All spectrophotometric analyses were performed on 3 biological replicates and analysed in 4 technical replicates. The statistically significant differences between digested and not digested infusion/decoction obtained for each plant tissues, were evaluated by *t*-test (*p<0.05* significance level). All statistical analyses were performed using R software version 3.5.1 (R Core Team, Vienna, Austria). The correlation indexes between total phenol contents and antioxidant activities were calculated using Microsoft Excel (Microsoft 365 MSO; 2307 version, Build 16.0.16626.20170). For the principal component analysis (PCA) of the ^1^H NMR spectra, RStudio version 1.4.1106 with Rnmr1D package [3] was used.

**Results and Discussion**

**Table S1** Minimum inhibitory concentration (MIC) values (mg/ml) of Hypericum perforatum and Borago officinalis not digested extracts against E. coli and S. aureus cells.

|  | ***H. perforatum* MIC** (mg/ml) | | ***B. officinalis* MIC** (mg/ml) | |
| --- | --- | --- | --- | --- |
| **Sample** | *E. coli* | *S. aureus* | *E. coli* | *S. aureus* |
| Flowers, infusion | 7.5 | 1.88 | 0.94 | 15 |
| Flowers, decoction | 0.94 | 0.94 | 0.47 | 15 |
| Leaves, infusion | 0.94 | 0.94 | 0.94 | 15 |
| Leaves, decoction | >15 | 0.94 | 3.75 | >15 |
| Stems, infusion | - | - | 7.5 | >15 |
| Stems, decoction | - | - | 1.88 | >15 |

The tested concentration was considered effective with an inhibition of at least 70% of bacterial growth. MIC values identified as >15 indicate samples without observed bacterial inhibitory activity in the tested concentration range.

**Table S2** Percentages of E. coli and S. aureus growth inhibition by the MIC concentrations (Table S1) of Hypericum perforatum and Borago officinalis not digested extracts.

|  | ***H. perforatum*** | | ***B. officinalis*** | |
| --- | --- | --- | --- | --- |
| **Sample** | *E. coli* | *S. aureus* | *E. coli* | *S. aureus* |
| Flowers, infusion | OD: 75.6 ± 7.7; MTT: 88.4 ± 17.2 | OD: 118.7 ± 10.2; MTT: 102.3 ± 10.1 | OD: 71.2 ± 1.1; MTT: n.c. | OD: 117.6 ± 3.9; MTT: 97.1 ± 13.1 |
| Flowers, decoction | OD: 80.3 ± 7.8; MTT: 66.3 ± 5.2 | OD: 122.0 ± 12.0; MTT: 94.3 ± 9.0 | OD: 82.2 ± 13.1; MTT: 72.6 ± 52.0 | OD: n.c.  MTT: 91.3 ± 1.1 |
| Leaves, infusion | OD: 71.1 ± 3.9; MTT: 79.7 ± 9.5 | OD: 120.4 ± 11.0; MTT: 107.2 ± 10.0 | OD: 67.4 ± 0.5; MTT: n.c. | OD: 88.7 ± 6.2; MTT: 92.7 ± 8.1 |
| Leaves, decoction | - | OD: 89.3 ± 47.9; MTT: 61.4 ± 48.0 | OD: 74.6 ± 4.2; MTT: 81.8 ± 3.7 | - |
| Stems, infusion | - | - | OD: 64.5 ± 6.0; MTT: 83.0 ± 3.0 | - |
| Stems, decoction | - | - | OD: 72.3 ± 5.0; MTT: 51.0 ± 4.8 | - |

OD, optical density (Abs_600nm_) measurement; MTT, measurement after addition of thiazolyl blue tetrazolium bromide reagent. Data are the mean ± SD (n = 3). n.c.: data not consistent with related OD or MTT value.


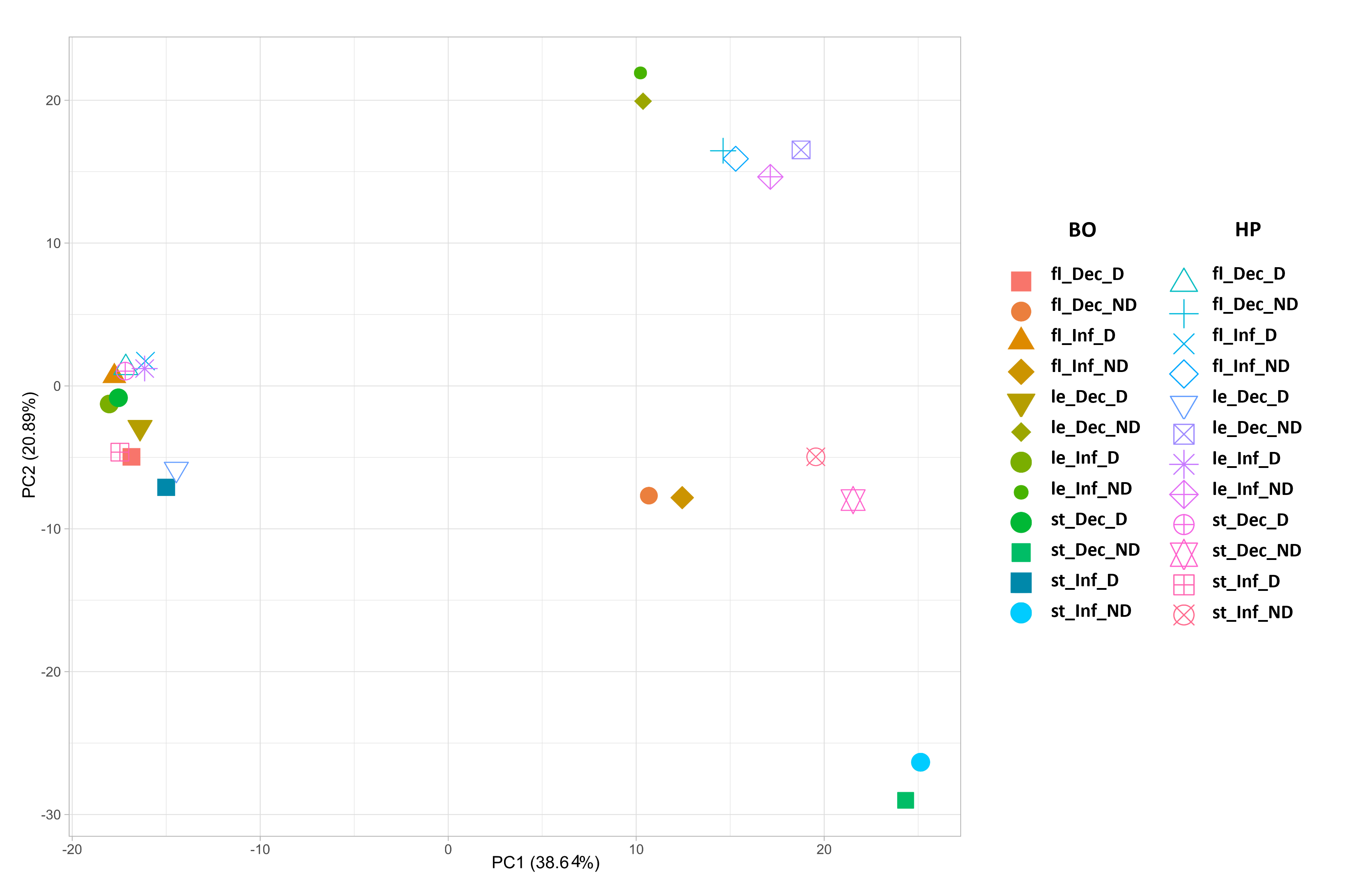


**Fig. S1** Principal component analysis (PCA) of all *Hypericum perforatum* (HP) and *Borago officinalis* (BO) NMR spectra (Rnmr1D package). i: infusion; d: decoction; ND: not digested; D: digested; fl: flowers; le: leaves; st: stems.


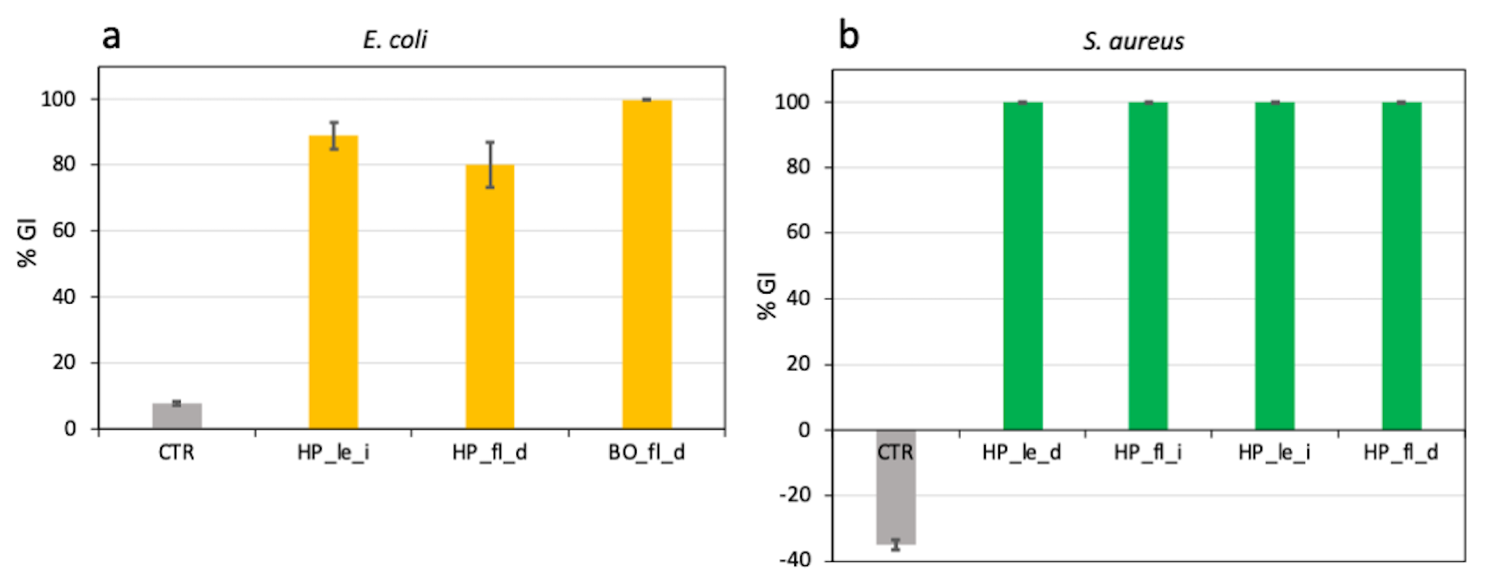


**Fig. S2** *Hypericum perforatum* (HP) and *Borago officinalis* (BO) not digested infusion and decoction extracts bactericidal activity against (**a**) *E. coli* and (**b**) *S. aureus* presented as the growth inhibition (GI %). CTR: assay without plant extract. Data are the mean ± SD (n = 3). i: infusion; d: decoction; le: leaves; fl: flowers.

**References for Materials and Methods**

1. Monari S, Ferri M, Salinitro M, Tassoni A (2023) New insights on primary and secondary metabolite contents of seven Italian wild food plants with medicinal applications: a comparative study. Plants 12(18):3180. <https://doi.org/https://doi.org/10.3390/plants12183180>

2. Brodkorb A, Egger L, Alminger M, Alvito P, Assunção R, Ballance S, et al. (2019) INFOGEST static *in vitro* simulation of gastrointestinal food digestion. Nat Protoc 14(4):991-1014. <https://doi.org/https://doi.org/10.1038/s41596-018-0119-1>

3. NMRProcFlow v1.4, in, NMRProcFLow n.d.

4. Ferri M, Gianotti A, Tassoni A (2013) Optimisation of assay conditions for the determination of antioxidant capacity and polyphenols in cereal food components. J Food Compos Anal 30(2):94-101. <https://doi.org/https://doi.org/10.1016/j.jfca.2013.02.004>

5. Bailey M, Biely P, Poutanen K (1992) Interlaboratory testing of methods for assay of xylanase activity. J Biotechnol 23:257-270. <https://doi.org/https://doi.org/10.1016/0168-1656(92)90074-J>

6. Lowry OH, Rosebrough NJ, Farr AL, Randall RJ (1951) Protein measurement with the Folin phenol reagent. J Biol Chem 193(1):265-275. <https://doi.org/https://doi.org/10.1016/S0021-9258(19)52451-6>

7. Monari S, Ferri M, Montecchi B, Salinitro M, Tassoni A (2021) Phytochemical characterization of raw and cooked traditionally consumed alimurgic plants. Plos One 16(8):e0256703. <https://doi.org/https://doi.org/10.1371/journal.pone.0256703>

8. Ferri M, Happel A, Zanaroli G, Bertolini M, Chiesa S, Commisso M, et al. (2020) Advances in combined enzymatic extraction of ferulic acid from wheat bran. New Biotechnol 56:38-45. <https://doi.org/https://doi.org/10.1016/j.nbt.2019.10.010>
